# Dosage of heparin for patency of the totally implanted central venous catheter in cancer patients

**DOI:** 10.1590/1518-8345.3326.3304

**Published:** 2020-06-19

**Authors:** Francisca Jane Gomes de Oliveira, Andrea Bezerra Rodrigues, Islane Costa Ramos, Joselany Áfio Caetano

**Affiliations:** 1 Universidade Federal do Ceará, Fortaleza, CE, Brazil.; 2 Hospital Monte Klinikum, Unidade de Terapia Intensiva, Fortaleza, CE, Brazil.; 3 Hospital Universitário Walter Cantídio, Centro Cirúrgico, Fortaleza, CE, Brazil.; 4 Universidade Federal do Ceará, Departamento de Enfermagem, Fortaleza, CE, Brazil.

**Keywords:** Vascular Access Devices, Central Venous Catheters, Catheters, Indwelling, Heparin, Heparin Lock, Catheter Obstruction, Dispositivos de Acesso Vascular, Cateteres Venosos Centrais, Cateteres de Demora, Heparina, Lock de Heparina, Obstrução do Cateter, Dispositivos de Acceso Vascular, Catéteres Venosos Centrales, Catéteres de Permanencia, Heparina, Cerradura de la Heparina, Obstrucción del Catéter

## Abstract

**Objective:**

to analyze the evidence available in the literature about the lowest necessary dose of heparin to maintain the patency of the totally implanted central venous catheter in adult cancer patients.

**Method:**

an integrative literature review, carried out in the following databases: *Literatura Latino-Americana e do Caribe em Ciências de Saúde*, Sciverse Scopus, Web of Science, Cumulative Index to Nursing and Allied Health Literature, Cochrane Central Register of Controlled Trials, including thirteen studies.

**Results:**

the evidence showed that the dose of heparin (300 IU/ml) is the most used in maintaining the patency of the totally implanted central venous catheter.

**Conclusion:**

according to the selected studies, the lowest dose of heparin found in maintaining the patency of the totally implanted central venous catheter in cancer patients was 10 UN/ml with a volume of 5 ml of the heparin solution.

## Abstract (pt)

**Objetivo:**

analisar as evidências disponíveis na literatura sobre a menor dose necessária de heparina para manter a patência do cateter venoso central totalmente implantado em pacientes oncológicos adultos.

**Método:**

revisão integrativa da literatura, realizada nas bases de dados: Literatura Latino-Americana e do Caribe em Ciências de Saúde, Sciverse SCOPUS, Web of Science, Cumulative Index to Nursing and Allied Health Literature, Cochrane Central Register of Controlled Trials, sendo incluídos treze estudos.

**Resultados:**

as evidências mostraram que a dose de heparina (300 UI/ml), é a mais utilizada na manutenção da patência do cateter venoso central totalmente implantado.

**Conclusão:**

de acordo com os estudos selecionados a menor dose de heparina encontrada na manutenção da patência do cateter venoso central totalmente implantado em pacientes oncológicos, foi de 10 UN/ml com um volume de 5 ml da solução de heparina.

## Abstract (es)

**Objetivo:**

analizar la evidencia disponible en la literatura sobre la dosis más baja de heparina necesaria para mantener la permeabilidad del catéter venoso central totalmente implantado en pacientes oncológicos adultos.

**Método:**

revisión integradora de la literatura realizada en las siguientes bases de datos: Literatura Latinoamericana y del Caribe en Ciencias de la Salud, *Sciverse Scopus*, *Web of Science*, *Cumulative Index to Nursing and Allied Health Literature*, *Cochrane Central Register of Controlled Trials*, con la inclusión de trece estudios.

**Resultados:**

las pruebas demostraron que la dosis de heparina (300 UI/ml) es la más utilizada para mantener la permeabilidad del catéter venoso central totalmente implantado.

**Conclusión:**

según los estudios seleccionados, la dosis más baja de heparina encontrada en el mantenimiento de la permeabilidad del catéter venoso central totalmente implantado en pacientes oncológicos fue de 10 UN/ml con un volumen de 5 ml de solución de heparina.

## Introduction

Among the options of devices used for the long-term administration of chemotherapy in cancer patients is the totally implanted central venous catheter (CVC-TI), such as the *port-a-cath* ^®^, a siliconized rubber device, surgically implanted, which has a reservoir located at the distal end, which remains below the skin in the thoracic region, on a bone surface^(^ 1 ^)^.

The CVC-TI offers greater comfort to the patient and a lower infection rate, reduces the risk of thrombosis, allows for outpatient treatment, does not interfere in the patient’s daily activities, and preserves the peripheral venous system, in addition to reducing the suffering and stress of the patients by avoiding repeated unsuccessful venous punctures, when compared to other available catheters^(^ 2 ^)^.

Although widely used, this device is not exempt from complications, such as hematomas, gas embolism, complications resulting from the anesthetic act, cardiac tamponade, and intolerance to the catheter. And because it is a long-term catheter, late complications are also added, such as: thrombosis, infection, catheter migration, rupture or fracture of the system, and catheter occlusion, among others^(^ 3 ^)^.

Occlusion of a CVC-TI is defined as the inability to infuse and/or draw blood from it, which can be classified as thrombotic, mechanical or chemical, being an event of concern for the health team, as it is mostly related to suspension of therapy or even exposure of the patient to a new invasive procedure^(^ 4 ^-^ 5 ^)^.

The Occlusion Management Guideline for Central Venous Access Devices (CVADs) guideline, whose purpose is to standardize the care related to the clinical practice in order to obtain positive results with a CVC-TI, considers the health professional fundamental for the management, prevention, and treatment of the occlusion, as this is the main responsible for its direct handling^(^ 6 ^)^.

Thus, in order to reduce complications related to this device, its handling, maintenance and optimization can be understood as a set of practices in which the nurse must gather knowledge, skills and attitudes that enable him to ensure an appropriate handling of them.

To guarantee CVC-TI patency, some precautions are necessary such as using the appropriate solution and performing the correct washing and blocking technique of this device, according to available protocols and guidelines, thus preventing its occlusion^(^ 4 ^,^ 7 ^)^.

A qualitative study on nurses’ knowledge related to the maintenance of the CVC-TI pointed out that one of the most frequent doubts refers to the ideal dose of heparin to maintain the patency of the device, and the time between each dose, when the catheter is not in continuous use. This corroborates the opinion of the authors, who state that, although there are several guidelines and rules related to this context, when addressing the solution and suggested dose to maintain CVC-TI patency, doubts still remain, as there are several practices in use in the clinical setting (saline solution, heparin, sodium citrate, among other chemical solutions)^(^ 8 ^-^ 10 ^)^.

Over the years, the heparinized solution has been the most used method to maintain the patency of the catheter; however, the routine of this technique seems to hide the iatrogenic effects of the drug itself, such as thrombocytopenia, which occurs due to its connection with an inhibitor of serine protease, antithrombin (AT), causing conformational change in the AT molecule, resulting in increased inhibition of thrombin (factor IIa) and of other serine proteases involved in the coagulation cascade. As thrombin stimulates the conversion of fibrinogen to fibrin, being inhibited, consequently, it generates a decrease in the formation of fibrin^(^ 11 ^)^.

Because it is an anticoagulant that acts at the level of the coagulation cascade and contributes to the development of adverse events, even if used in small quantities, such as washing (flush) of central venous catheters, it affects up to 20 to 30% of the patients who are exposed to the drug^(^ 12 ^-^ 13 ^)^. In this context, the objective of this study is to analyze scientific evidence in the literature on the lowest necessary dose of heparin to maintain the patency of the totally implanted central venous catheter in adult cancer patients.

## Method

This is an integrative literature review study, which allows for research studies already carried out to be summarized and conclusions to be established based on the critical evaluation of different methodological approaches, aiming to synthesize and analyze the data to develop a more comprehensive explanation of a specific phenomenon from the synthesis or analysis of study findings, with theoretical and/or interventionist purposes^(^ 14 ^)^.

Thus, six stages were adopted for the elaboration of this review: selection of the research question; definition of study inclusion criteria and sample selection; representation of the selected studies in table format, considering all the characteristics in common; critical analysis of the findings, identifying differences and conflicts; interpretation of the results, and clearly reporting the evidence found^(^ 15 ^)^.

For the elaboration of the guiding question, the PICOS strategy was used, with P for Population, Patient or Problem (adult cancer patients with totally implanted central venous catheter), I for Intervention or area of Interest (heparin dose used in the maintenance of the CVC-TI), C for Comparison (heparin dose used to maintain the CVC-TI), O for Outcomes (maintenance of CVC-TI patency) and S for the type of Studies used (systematic reviews of controlled and randomized clinical trials, controlled and randomized clinical trials, systematic review of cohort studies, and cohort studies)^(^ 16 ^)^. Thus, this study sought to answer the following question: What evidence is available in the literature about the lowest necessary dose of heparin to maintain the patency of the totally implanted central venous catheter in adult cancer patients?

As an inclusion criterion, studies published in English, Spanish or Portuguese were chosen, which included the thematic use of heparin to maintain the CVC-TI in adult cancer patients, such as systematic reviews of controlled and randomized clinical trials, controlled and randomized clinical trials, systematic review of cohort studies, and cohort studies.

This criterion was established as a result of the answer to be obtained in relation to the guiding question of the study, in which it was sought to follow the classification of level of evidence proposed by the Oxford Centre for Evidence-Based Medicine, where the levels of evidence are classified as follows: 1A - systematic review (with homogeneity) of controlled and randomized clinical trials, 1B - controlled and randomized clinical trial with a narrow confidence interval, 1C - all-or-nothing therapeutic results, 2A - systematic review of cohort studies, 2B - cohort study, 2C - observational study, 3A - systematic review (with homogeneity) of case-control studies, 3B - case-control study, 4 - lower quality case series and cohort studies, and 5 - specialists’ opinion devoid of critical assessment, based on consensus, physiological studies^(^ 17 ^)^.

The search for the studies was carried out during the months of August and September 2018, in the following databases: Latin American and Caribbean Health Sciences Literature (*Literatura Latino-Americana e do Caribe em Ciências de Saúde*, LILACS), SCOPUS, Web of Science, Cumulative Index to Nursing and Allied Health Literature (CINAHL), and Cochrane Central Register of Controlled Trials (COCHRANE).

For the selection of the articles, a consultation was first made with the Health Science Descriptors (*Descritores em Ciência da Saúde*, DeCS) and the Medical Subject Headings (MeSH), in all the databases, with the following descriptors and their synonyms being identified and used with the use of the OR Boolean operator (MESH): “catheters, indwelling” OR “vascular access devices” OR “port catheters” OR “port a cath” OR “catheters, port” OR “port, vascular access” OR “vascular access port” OR “central venous catheters” OR “totally implantable venous device” OR “totally implantable central venous access port” OR heparin OR “heparin lock” OR “heparin flush” OR “obstruction catheter” OR “catheter obstruction”. Most of the descriptors were enclosed in quotation marks because they are compound terms.

After searching the databases, all the studies were sent to the EndNote X8 reference manager, where filtering was performed to exclude duplicate articles, being considered only once. Then, all the titles were read, and then the abstracts. After the final selection, the articles were read in full in order to select those who answered the guiding question of the research. After this process, publications that did not comply with the purpose of the study were excluded.

For the synthesis of the selected articles, an instrument was used that contemplates the following items: name of the article, authors, year of publication, level of evidence, objective, method, methodological quality and results, with the purpose of extracting, organizing and summarizing the information and facilitating the formation of the database.

Regarding the evaluation of the methodological quality of the randomized clinical trials, the scale proposed by Jadad, et al. (1996)^(^ 18 ^)^ was used. This scale consists of five criteria and varies from 0 to 5 points, where each item receives 1 point for the “yes” answer, or zero points for the “no” answer, in which a score below 3 indicates that the study has a low methodological quality and that its results can hardly be extrapolated to other scenarios.

The systematic reviews were assessed according to the Assessment of Multiple Systematic Reviews (AMSTAR). AMSTAR was built from the analysis and updating of other empirically validated instruments^(^ 19 ^)^. The items that comprise the checklist present minimum requirements for a systematic review: The review design was presented *a priori*? Was there duplication in data extraction and study selection? Was a comprehensive search of the databases carried out? Was the status of the publication (e.g., theses and dissertations, book chapters, etc.) used as an inclusion criterion? Has a list of included and excluded studies been provided? Were the characteristics of the included studies provided? Has the quality of the included studies been assessed and documented? Has the quality of the included studies been used appropriately in the conclusions? Were the methods used to group the findings of the included studies appropriate? Has publication bias been assessed? Has the conflict of interest been described? For each item of the instrument there are two answer options: a) “yes”, if the review explicitly contemplates the criterion; b) “no”, if it does not. For each “yes” answer, one point^(^ 20 ^)^ is applied. The more items in accordance with the checklist, the better the methodological quality of the study. The calculation of the final score was converted into a percentage and the quality of the manuscripts was assessed in three categories for both criteria: A for studies that meet more than 80% of the criteria established by Strengthening the Reporting of Observational Studies in Epidemiology (STROBE); B for studies that meet 50% to 80% of these criteria; and C for studies that meet less than 50% of the criteria^(^ 21 ^)^.

The STROBE guidelines were used to assess the observational studies, in which a score of 0 means “does not meet” and a score of 1 means “meets”, being used for each of the 22 items of the STROBE guidelines. As well as in AMSTAR, the calculation of the final score was converted into a percentage and the quality of the manuscripts was assessed in three categories for both criteria: A for studies that meet more than 80% of the criteria established by STROBE; B for studies that meet 50% to 80% of these criteria; and C for studies that meet less than 50% of the criteria^(^ 21 ^)^.

Independently, two researchers carried out the selection of studies: first, studies were excluded from the reading of their titles (first analysis), then abstracts (second analysis) and, finally, after reading the full texts (third analysis). In case of disagreement or doubt, a third experienced researcher was consulted.

The discussion of the obtained data was carried out in a descriptive way, allowing the reader to evaluate the applicability of the integrative review elaborated, in order to achieve the objective of this method, that is, to facilitate the incorporation of evidence and build knowledge in the area of higher education in Nursing.

## Results

**Figure 1:**
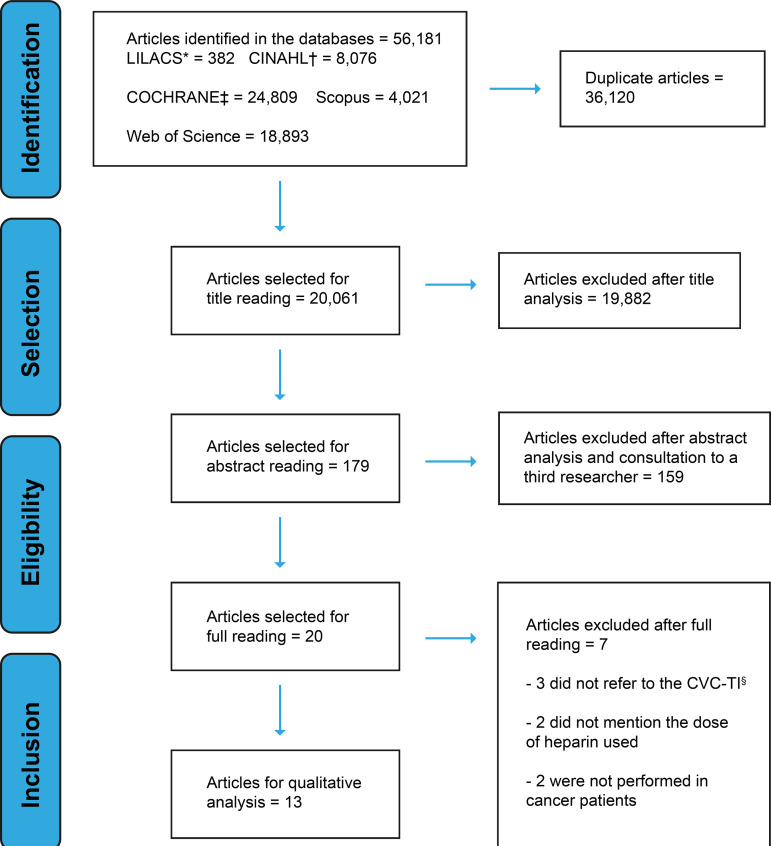
Flowchart of the selection process for the primary studies. Fortaleza, CE, Brazil, 2018 ^*^LILACS = Literatura Latino-Americana e do Caribe em Ciências de Saúde; ^†^CINAHL = *Cumulative Index to Nursing and Allied Health Literature*; ^‡^COCHRANE = *Cochrane Central Register of Controlled Trials*, ^§^CVC-TI = Totally Implanted Central Venous Catheter

At the end of the article search process, the sample of the integrative review was composed of 13 primary studies, as shown in Figure 1.

Of the 13 selected articles, 11 were considered of high methodological quality, and two of medium methodological quality, according to the adopted criteria. The E07 article was evaluated with medium methodological quality, for meeting 6/11 evaluated items (55%), and the E09 study with high methodological quality for meeting 11/11 evaluated items (100%).

As for the characteristics of the methodological designs, seven were cohort studies (E01, E03, E05, E06, E07, E10 and E12), four randomized clinical trials (E02, E04, E11, and E13) and two systematic reviews (E08, E09).

With regard to the study site, two were developed in the United States of America (E01, E06), four in Italy (E02, E5, E10, E13), two in Belgium (E4, E8), one in Brazil (E03), one in Iraq (E07), one in the United Kingdom (E09), one in Sweden (E11) and one in Istanbul (E12). The characteristics of the studies, with regard to the identification code, authors, title and level of evidence, are represented in Figure 2.

**Figure 2:** Distribution of the primary studies according to author, title, level of evidence and methodological quality. Fortaleza, CE, Brazil, 2018

Study code/Author(s)/ Year	Title	Evidence level	Methodological quality
E01. Girda E; Phaeton R; Nevadunsky N; Huang G; Smith Ho; Smotkin D; Goldberg G; Kuo D, 2013^(^ 1 ^)^.	Extending the interval for port-a-cath maintenance	2B*	Category A^§^
E02. Dal Molin A, Clerico M, Baccini M, Guerretta L, Sartorello B, Rasero L, 2015^(^ 22 ^)^.	Normal saline versus heparin solution to lock totally implanted venous access devices: Results from a multicenter randomized trial	1B^†^	High quality
E03. Brito, 2018^(^ 23 ^)^.	Comparison between Saline Solution Containing Heparin versus Saline Solution in the Lock of Totally Implantable Catheters	2B*	Category A^§^
E04. Goossens GA; Jérôme M; Janssens C; Peetermans WE; Fieuws S; Moons P; Verschakelen J; Peerlinck K; Jacquemin M; Stas M, 2013^(^ 24 ^)^.	Comparing normal saline versus diluted heparin to lock non-valved totally implantable venous access devices in cancer patients: a randomised, non-inferiority, open trial	1B ^†^	High quality
E05. Bertoglio S, Solari N, Meszaros P, Vassallo F, Bonvento M, Pastorino S, Bruzzi P, 2012^(^ 25 ^)^.	Efficacy of normal saline versus heparinized saline solution for locking catheters of totally implantable long-term central vascular access devices in adult cancer patients	2B*	Category A^§^
E06.Kuo YS; Schwartz B; Santiago J; Anderson PS; Fields AL; Goldberg GL, 2005^(^ 26 ^)^.	How Often Should a Port-A-Cath be Flushed?	2B*	Category B^||^
E07. Baram A, Majeed G, Abdullah H, Subhi A, 2014^(^ 27 ^)^.	Heparin versus Saline Solution for Locking of Totally Implantable Venous Access Port (TIVAP): Cohort Study of the First Kurdistan Series of TIVAP	2B*	Category A^§^
E08. Goossens GA, 2014^(^ 28 ^)^.	Flushing and Locking of Venous Catheters: Available Evidence and Evidence Deficit	1A^‡^	Medium quality
E09. López-Briz E, Ruiz GV, Cabello JB, Bort-Marti S, Carbonell SR, Burls A, 2014^(^ 29 ^)^.	Heparin versus 0.9% sodium chloride intermittent flushing for prevention of occlusion in central venous catheters in adults	1A^‡^	High quality
E10. Palese A, Baldassar D, Rupil A et al. 2014^(^ 3 ^)^.	Maintaining patency in totally implantable venous access devices (TIVAD): A time-to-event analysis of different lock irrigation intervals	2B*	Category A^§^
E11. Johansson E, Björkholm M, Björvell H et al. 2004^(^ 30 ^)^.	Totally implantable subcutaneous port system versus central venous catheter placed before induction chemotherapy in patients with acute leukaemia-a randomized study	1B^†^	High quality
E12. Kefeli U, Dane F, Yumuk PF, et al. 2009^(^ 31 ^)^.	Prolonged interval in prophylactic heparin flushing for maintenance of subcutaneous implanted port care in patients with cancer	2B*	Category A^§^
E13. Biffi R, Braud F, Orsi F et al. 2001^(^ 32 ^)^.	A randomized, prospective trial of central venous ports connected to standard open-ended or Groshong catheters in adult oncology patients	1B^†^	High quality

Classification of the level of evidence of the studies according to the Oxford Center for Evidence-Based Medicine:

* 2B = cohort studies;

† 1B = studies of controlled and randomized clinical trial with a narrow confidence interval;

‡ 1^st^= systematic review studies (with homogeneity) of controlled and randomized clinical trials. Evaluation of the methodological quality:

§ Category A = studies evaluated according to the criteria of the Strengthening the Reporting of Observational Studies in Epidemiology (STROBE) of the Assessment of Multiple Systematic Reviews (AMSTAR) and that met more than 80% of the established criteria;

|| Category B = studies evaluated according to the criteria of the Strengthening the Reporting of Observational Studies in Epidemiology (STROBE) or the Assessment of Multiple Systematic Reviews (AMSTAR) and that met 50% to 80% of these criteria

Regarding the objectives of the studies, five evaluated the efficacy of the saline solution compared to heparin to maintain the patency of the catheter (E02, E03, E04, E05, E07, E09), five sought to know a longer interval for maintaining the CVC-TI (E01, E06, E10, E11, E12), two compared the effectiveness of the catheters and their complications in cancer patients (E11, E13) and one evaluated flushs and locks for maintaining CVC-TI patency (E08). Figure 3 shows the description of the articles selected for the study, with regard to the objective, intervention and results.

**Figure 3:** Synthesis of the primary articles according to the objective, intervention and results. Fortaleza, CE, Brazil, 2018

Objective	Intervention/Maintenance	Result
E01. To standardize a safe and adequate interval to maintain CVC-TI patency*.	The protocol used a 10 ml flush^†^ of physiological serum followed by a 5 ml heparin lock (100 IU/ml^‡^). Total number of patients included: 201.	When compared to 90-day maintenance versus those more than 90 days apart (mean of 112 days), there was no difference in occlusion rates between the groups.
E02. To determine the effectiveness of the saline solution compared to heparin in maintaining totally implanted venous access devices.	Saline Solution Group (203 patients): flush with 20 ml^†^saline solution, followed by a block with 5 ml^†^ saline solution, using positive pressure. Heparin Group (212 patients): wash with 10 ml^†^saline solution followed by a block with 5 ml^†^heparin (10 IU/ml^‡^).	CVC-TI* occlusions were observed in 24 patients: 10 (4.71%) in heparin and 14 (6.90%) in the normal saline group, with no significant difference in the results.
E03. To compare the heparinized saline solution versus 0.9% saline solution for the maintenance of CVC-TI*.	Heparin Group (270 patients): maintenance consisting of 1.5 ml^†^of 0.9% saline solution with heparin content (100 IU/ml^‡^). Saline Solution Group (592 patients): maintenance with 1.5 ml^†^of 0.9% saline solution.	Regarding CVC-TI* occlusion, there were 8 cases in the Heparin group and 8 cases in the Saline Solution group, with no statistical difference between the groups.
E04. To evaluate the effectiveness of heparin compared to the saline solution.	Saline Solution Group (404 patients): wash with 10 ml^†^saline solution before and after blood collection and drug administration, every 8 weeks, when the device was not in use and with 20 ml^†^saline solution after administration of blood (components) or parenteral nutrition. Heparin Group (398 patients): block with 3 ml^†^heparin before the needle is removed.	No significant complications were found when using saline solution instead of heparin as a blocking solution for catheter maintenance.
E05. To evaluate the efficacy and safety of the normal saline solution for CVC-TI blocking procedures*.	Heparin Group (297 patients): wash with heparinized solution (500 IU/10 ml^‡^) Saline Solution Group (313 patients): 10 ml^†^normal saline solution.	The results do not show statistically significant differences with regard to catheter obstruction.
E06. To demonstrate that a longer maintenance interval for CVC-TI* can be safe, convenient, and more efficient.	Washing the catheter with 10 ml^†^saline solution followed by a block with 5 ml^†^heparin (100 IU/ml^‡^). Total number of patients included: 82.	The mean intervals for catheter maintenance ranged from 38 to 244 days, with a mean interval between patients without complications associated with catheter obstruction of 63 days.
E07. To evaluate the efficacy and safety of the normal saline solution in the practice of maintaining CVC-TI* in cancer patients	Heparin Group (194 patients): wash twice a month with heparinized solution (20 ml^†^normal saline solution and 5,000 IU/ml^‡^ unfractionated heparin). Saline Solution Group (190 patients): wash the catheter with 20 ml^†^saline solution twice a month.	The incidence of catheter-related occlusion was quite low for both groups, with no significant differences between the two groups.
E08. To clarify issues related to washing and blocking the CVC-TIs* and to describe the available evidence regarding the benefits of the interventions in relation to occlusion.	Washing and blocking venous catheters.	For washing the catheter a volume of 10 ml^†^saline solution is sufficient. Regarding the block, volumes should be minimal and based on the prime of the catheter. A maximum of 1 ml^†^excess volume for the block is adequate to safely fill the catheter and any complements.
E09. To evaluate the effectiveness of washing with heparin versus saline solution in adults with central venous catheters.	Heparin x saline solution.	The review found no convincing evidence of a reduction in the CVC^§^ occlusion rate maintained with heparin compared to CVC^§^ maintained with sterile saline solution. As heparin is more expensive, the results of this review do not support its use, except in future clinical trials.
E10. To evaluate the efficacy of irrigating CVC-TI*devices every eight weeks, instead of every four weeks, to maintain the patency of the device.	A wash with 20 ml^†^normal saline solution, followed by a block with 3 ml^†^sodium heparin (250 IU/5 ml^‡^) for a total of 150 units of heparin was performed with two homogeneous groups with catheter maintenance every 4 weeks (17 patients) and 8 weeks (20 patients).	There were no differences in the occurrence of occlusion between CVC-TIs* irrigated every four weeks, instead of every eight weeks.
E11. To compare the survival time, function, and complication rates of double lumen CVC^§^ use versus CVC-TI* for chemotherapy in patients with leukemia.	CVC-TI Group* (19 patients): wash with 5 ml^†^heparinized saline solution (100 IU/ml^‡^) after each use or at least once a month. Double lumen CVC^§^ (24 patients): wash with 5 ml^†^heparinized saline solution (12.5 IU/ml^‡^) after use and at least twice a week.	There was no significant difference between the two groups regarding the catheter survival time. CVC^§^ occlusion was noted on 14 occasions in seven patients; and CVC-TI* occlusion, in 3 patients.
E12. To compare the safety and efficacy of administering a larger dose of heparin (1,000 IU/ml^‡^) and flushes every 6 weeks, versus standard dose and schedule (500 IU/ml^‡^ every 4 weeks), to reduce the incidence of CVC-TI*-related infections and thrombosis.	For catheter maintenance after chemotherapy. Group 1 (59 patients): they received 1,000 IU/ml^‡^heparin in 3 ml^†^normal saline solution every 6 weeks. Group 2 (30 patients): they received 500 IU/ml^‡^heparin in 3.5 ml^†^normal saline solution every 4 weeks.	Maintaining catheter patency with 1,000 IU/ml^‡^heparin every 6 weeks may be a safer, easier, more economical, comfortable and effective alternative when compared to standard 4-week administration to prevent thrombosis and infections.
E13. To compare the associated complications in patients with Groshong catheter and CVC-TI* in cancer patients undergoing chemotherapy.	Both the control (152 patients with Groshong catheter) and the intervention (152 patients with CVC-TI*) groups received 5 ml^†^heparin (50 IU/ml^‡^) for catheter maintenance every 28 days.	It has been shown that the Groshong central venous catheter (at least when used for the administration of long-term chemotherapy) is not superior to CVC-TI* in terms of early and late complications. With regard to catheter obstruction, there was no difference between the two.

* CVC-TI = Totally Implanted Central Venous Catheter;

† ml = Milliliters; <

‡ International units per milliliter;

§ CVC = Central Venous Catheter

Regarding the concentration and volume of heparin used to maintain CVC-TI patency, seven studies (E01, E03, E04, E06, E08 , E09, E11) used a heparin concentration of 100 IU/ml with the administered volume ranging between 3 and 5 ml. Higher concentrations of heparin (500 IU/ml, 5,000 IU/ml, 250 IU/ml and 1,000 IU/ml) were identified in studies E05, E07, E10, and E12 which, when compared to the saline solution for CVC-TI maintenance, and either this procedure was performed with an interval of 15 to 20 days (E07, E02), 28 days (E03, E05, E09, E11, E13) or with intervals longer than 56 to 90 days (E04, E08, E01, E10, E12), no significant differences were identified with regard to obstruction or other complications associated with the catheter. Figure 4 shows the concentrations and volume of heparin used in studies for maintaining CVC-TI and the interval between the applications.

**Figure 4:** Synthesis of the studies according to concentration/ml, volume, total heparin concentration, and maintenance interval. Fortaleza, CE, Brazil, 2018

Studies	Heparin concentration (IU/ml*)	Heparin volume	Total heparin concentration	Maintenance interval
E 01	100 IU/ml*	5 ml^†^	500 IU^§^	90 days
E 02	10 IU/ml*	5 ml^†^	50 IU^§^	20 days
E 03	100 IU/ml*	1.5 ml^†^	150 IU^§^	28 days
E 04	100 IU/ml*	3 ml^†^	300 IU^§^	56 days
E 05	500 IU/ml*	10 ml^†^ of the solution^‡^	500 IU^§^	28 days
E 06	100 IU/ml*	5 ml^†^	500 IU^§^	38 days
E 07	5000 IU/ml*	20 ml^†^ of the solution^‡^	5000 IU^§^	15 days
E 08	100 IU/ml*	2.5 ml^†^	250 IU^§^	42-56 days
E 09	100 IU/ml*	3 ml^†^	300 IU^§^	28 days
E 10	250 IU/5 ml*	3 ml^†^	750 IU^§^	28-56 days
E 11	100 IU/ml*	5 ml^†^	500 IU^§^	28 days
E 12	1,000 IU/ml* and 500 IU/ml*	3 ml^†^ and 3.5 ml^†^	3000 IU^§^ and 1,750 IU^§^	28 and 42 days
E 13	50 IU/ml*	5 ml^†^	250 IU^§^	28 days

* IU/ml = International units *per* milliliter;

† ml = Milliliters;

‡ solution = Solution composed of the heparin concentration reported in the table, combined with this quoted volume of saline solution;

§ IU = International units

## Discussion

Obstruction of the CVC-TI results in suspension of treatment, increased risks and related costs, making it a relevant concern for the health professionals. Therefore, measures to reduce this problem are of crucial importance, especially with regard to the choice of the solution and the dose to be used to maintain the patency of the device^(^ 9 ^)^.

Most of the studies (E02, E03, E04, E05, and E07) compared the effectiveness of the saline solution versus heparin for maintaining CVC-TI patency, showing that there are no significant complications when using the saline solution instead of heparin as a catheter block solution. Thus, it is believed that, for the decision to use the heparinized solution or 0.9% sodium chloride, a critical analysis based on scientific evidence is necessary, an essential tool for the promotion of quality care and consequent gain of gains in health.

According to the literature, the conclusion that the saline solution is as effective as the heparin solution for maintaining CVC-TI patency seems to be well supported, as it prevents the occurrence of adverse events resulting from the use of heparin, such as thrombocytopenia heparin-induced hemorrhage, among others already mentioned^(^ 33 ^)^

Regarding the dose of heparin necessary to maintain CVC-TI patency, studies E01, E04, E06, E09, E11, and E12 show that the concentrations ranged from 10 IU/ml to 5,000 IU/ml, with a predominance of the concentration of 100 IU/ml, with a volume ranging from 3 to 5 ml of the CVC-TI blocking solution.

The dose of heparin required to maintain CVC-TI patency can vary from 10 to 1,000 IU/ml, with the concentration of 100 IU/ml in a volume of 3 ml being the most commonly used^(^ 2 ^,^ 31 ^,^ 22 ^,^ 34 ^)^. This recommendation corroborates the results of Goossens, et al. 2015, and the recommendations from the Infusion Nursing Society (INS) (2016), which indicate the use of the lowest possible heparin concentration to maintain catheter patency^(^ 28 ^,^ 35 ^)^.

A retrospective cohort study, performed with 2,996 patients with breast cancer, at the Breast Disease Center of the 4^th^ Hospital of the Hebei Medical University in China, used a heparin concentration of 100 IU/ml to maintain CVC-TI patency, presenting an occlusion rate of 4.3%, with maintenance every 28 days^(^ 36 ^)^.

Although 40% of the selected studies used 5 ml of the solution as a blocking volume for the CVC-TI and 31% only 3 ml of the solution, it is important to note that the volume of the block used for maintaining the CVC-TI must be at least two times the prime of the device used in question, thus avoiding incomplete filling which, consequently, would increase the risk of obstruction^((31,22, 35))^.

In spite of the variation in the solution dose, the CVC-TI reservoir should be considered, which has a dead space and an internal volume larger than a standard catheter. Thus, a volume of 2.5 ml to 3 ml is used to perform the catheter block, so that the amount is slightly higher than the prime of the catheter, considering that the adherence of lipids, fibrin, and other drug deposits to the reservoir wall can result in the colonization of microorganisms and subsequently in bloodstream infection related to the catheter^(^ 2 ^,^ 28 ^)^.

A study carried out in the National Cancer Institute (*Instituto Nacional de Câncer*, INCA) involving 69 CVC-TI implantations, taking into account the length of the catheter inserted in the patients and the necessary volume of the solution for its filling, found that the volume required for filling the catheter, varies from 0.62 ml to 1 ml and that, according to the INS recommendations, the volume of the filling solution should vary from 1.24 ml to 2 ml^(^ 35 ^,^ 37 ^)^.

Regarding the time interval for maintaining the patency of the device, for studies E03, E05, E09, E10, E11, E12, and E13, an interval of 28 to 56 days was observed. However, it is worth mentioning that it is feasible to extend the CVC-TI maintenance period, without harming the patient, aiming at improving their quality of life and reducing hospital costs.

INS (2017) emphasizes that the CVC-TIs should be washed after each use (infusion of drugs, serum, blood, among others) and, when not in use, the manufacturer’s recommendations should be taken into account if the institution does not have an infusion therapy team and/or protocols, or every four weeks, classifying this recommendation as IIIC. It should be noted that studies on the permeability of the CVC-TI are still scarce^(^ 38 ^-^ 40 ^)^.

Several research studies suggest that the monthly maintenance of the CVC-TIs is excessive, inconvenient for patients, and expensive. Thus, some centers have already started the practice of washing the CVC-TIs once every 90 days, when they are not in use, ensuring that they are effective, safe and capable of increasing patient compliance and satisfaction, in addition to reducing costs both for patients and for health care systems^(^ 24 ^,^ 41 ^-^ 42 ^)^.

When using a concentration of 100 IU/ml (3 ml), seeking to extend the CVC-TI wash interval to 56 to 90 days, there were no changes in relation to catheter obstruction, regarding the maintenance performed every 28 days, presenting an occlusion rate of up to 3%^(^ 24 ^,^ 42 ^)^.

The maintenance of a CVC-TI has a mean cost of US$ 160.00, including time and nursing equipment. Thus, reducing the number of washes from 12 to 4 times a year would reduce the annual cost from US$ 1,920.00 to US$ 640.00. This is without considering time off work, transportation costs, and other logistical expenses incurred by the patient. The considerable reduction in individual costs for patients has an even more significant economic impact when considered at a population level^(^ 36 ^)^.

The Centers for Disease Control and Prevention (CDCs) and the Oncology Nurse Society guidelines provide some evidence of flexibility for the flush, when recommending a heparin solution of 100 IU/ml, 5 ml every month or every 6 to 8 weeks, and after each use of the device, but no recommendation regarding the ideal range^(^ 26 ^,^ 43 ^-^ 44 ^)^.

With regard to the limitation of this study, a lack of studies within this perspective was identified. This emphasizes the need to conduct clinical trials comparing the use, not only of different doses of heparin with each other, but also the use of physiological solution to maintain CVC-TI patency because, in addition to being free of complications, there is evidence that shows similar efficacy between the physiological solution and the heparin solution in maintaining the patency of the device, visualizing a safe and evidence-based care practice.

## Conclusion

This study allowed us to conclude that the dose of heparin most used to maintain the patency of the totally implanted central venous catheter in cancer patients was 100 UN/ml, with a volume ranging from 5 ml to 3 ml of the heparin solution, being the dose of 10 UN/ml the lowest dose found in maintenance, with an interval of 28 days between maintenance instances. However, it was possible to identify that there is an interest in extending the device’s heparinization interval, in addition to the use of saline solution to maintain this type of catheter.

As a contribution to the care practice, it is believed that this study presents evidence that lower doses of heparin are sufficient to maintain the previous CVC-TI, showing that doses higher than 300 UN/ml are unnecessary, in addition to being able to contribute to the development complications associated with its use, as previously mentioned.

It is important to note that clinical trials have been carried out in order to compare the effectiveness of heparin in relation to the physiological solution for maintaining CVC-TI patency, and have shown similar effects with regard to its obstruction rate, evidencing a field to be explored through new studies that seek to prove the effectiveness of the physiological solution.
